# METTL3 facilitates immunosurveillance by inhibiting YTHDF2-mediated NLRC5 mRNA degradation in endometrial cancer

**DOI:** 10.1186/s40364-023-00479-4

**Published:** 2023-04-21

**Authors:** Lei Zhan, Jing Zhang, Jun-Hui Zhang, Xiao-Jing Liu, Bao Guo, Jia-Hua Chen, Zhen-Hai Tang, Wen-Yan Wang, Qing-Yuan Wang, Bing Wei, Yun-Xia Cao

**Affiliations:** 1 Department of Obstetrics and Gynecology, The First Affiliated Hospital of Anhui Medical University, No 218 Jixi Road, Hefei, 230022 Anhui China; 2 Department of Obstetrics and Gynecology, The Second Affiliated Hospital of Anhui Medical University, No 678 Furong Road, Hefei, 230601 Anhui China; 3 NHC Key Laboratory of Study on Abnormal Gametes and Reproductive Tract, Anhui Medical University), No 81 Meishan Road, Hefei, 230032 Anhui China; 4 Key Laboratory of Population Health Across Life Cycle (Anhui Medical University), Ministry of Education of the People’s Republic of China, No 81 Meishan Road, Hefei, 230032 Anhui China; 5 Anhui Province Key Laboratory of Reproductive Health and Genetics, No 81 Meishan Road, Hefei, 230032 Anhui China; 6 Anhui Provincial Engineering Research Center of Biopreservation and Artificial Organs, No 81 Meishan Road, Hefei, 230032 Anhui China; 7 Anhui Provincial Institute of Translational Medicine, No 81 Meishan Road, Hefei, 230032 Anhui China; 8 Center for Scientific Research of Anhui Medical University, No 218 Jixi Road, Hefei, 230022 Anhui China

**Keywords:** Endometrial cancer, N6-methyladenosine, N6-adenosine-methyltransferase-like 3, YTH domain-containing family 2, NLR family CARD domain-containing 5, Immunosurveillance, Biomarker, Immunotherapy

## Abstract

**Background:**

N6-methyladenosine (m6A) methylation is the most abundant chemical posttranscriptional modification of mRNA, and it is associated with the regulation of the immune response to tumors. However, the function of m6A modification in the immune response to endometrial cancer (EC) remains unknown. Our study investigated the immunological role of methyltransferase-like 3 (METTL3) in EC and the underlying molecular mechanism.

**Methods:**

We investigated the correlation between the expression of METTL3 and CD8 by using an endometrial tissue microarray cohort. Next, we investigated the role and mechanism of METTL3 in the immune response to EC using a mouse tumor model and a CD8^+^ T cell-EC cell coculture system after METTL3 overexpression or depletion. Additionally, RNA immunoprecipitation (RIP), methylated RIP, and RNA stability experiments were used to investigate the mechanism underlying the function of METTL3 in immunosurveillance of EC.

**Results:**

METTL3 levels were downregulated in EC patients, low levels of METTL3 were correlated with poor prognosis in EC patients. There was a positive correlation between METTL3 expression and CD8 expression. Overexpression of METTL3 in the EC cell and CD8^+^ T cell coculture system inhibited EC cell proliferation, migration, and promoted CD8^+^ T-cell proliferation, and in vivo, METTL3 overexpression increased CD8^+^ T cell proportions and inhibited EC progression; however, genetic depletion of METTL3 exerted the opposite effects. NLR family CARD domain-containing 5 (NLRC5) was identified as a target of METTL3-mediated m6A modification. The degradation of NLRC5 was increased by YTH domain-containing family 2 (YTHDF2).

**Conclusions:**

Overall, METTL3, YTHDF2, and NLRC5 have potential to be the diagnostic and prognostic biomarkers for EC. METTL3 facilitated the m6A modifications of NLRC5 and inhibited its degradation through a YTHDF2-dependent mechanism in EC. Genetic overexpression of METTL3 attenuated the immune evasion of EC by promoting NLRC5-mediated immunosurveillance, suggesting that the METTL3/YTHDF2/NLRC5 axis is a promising target of immunotherapy in EC.

**Supplementary Information:**

The online version contains supplementary material available at 10.1186/s40364-023-00479-4.

## To the editor

The initiation and progression of EC are complicated processes, and epidemiological risk factors and genetic risk factors are well known to be responsible for the development of EC [1]. In recent years, tumor cell escape from immunosurveillance has been demonstrated to be a crucial mechanism underlying tumor development, including EC development [2]. However, the exact molecular mechanism underlying immune evasion in EC remains unclear. m6A modifications are the most prevalent modifications in human mRNA, and they perform vital functions in the pathophysiology of a wide range of diseases [3]. Previous studies have suggested that m6A could play an essential role in the progression of EC by regulating ion [4, 5].

**Fig. 1:**
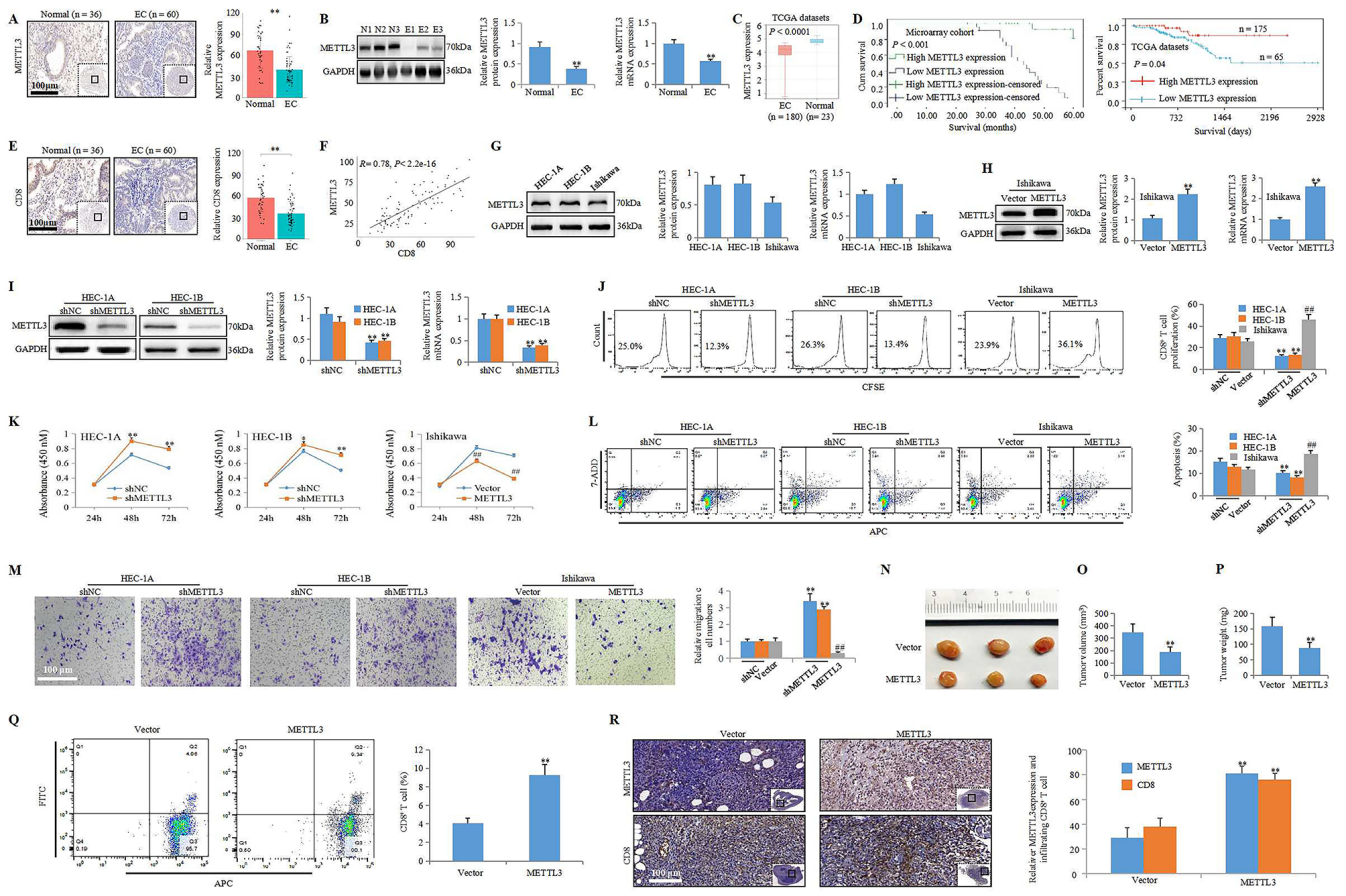
METTL3 inhibits EC by promoting immunosurveillance. **A** The endometria from endometrial tissues microarray cohort was subjected to IHC analysis to determine METTL3 level (scale bar: 100 μm; **P < 0.01 vs. Normal group). **B** The protein and mRNA expressions of METTL3 in endometria from normal subjects and EC patients (n = 3, **P < 0.01 vs. Normal group). **C** METTL3 expression in EC form TCGA datasets (****P < 0.0001 vs. Normal group). **D** Kaplan-Meier curves of correlation of METTL3 with the EC cumulative survival from endometrial tissues microarray cohort (*P* = 0.04) and TCGA datasets (*P* < 0.001). **E** The endometria from endometrial tissues microarray cohort was subjected to IHC analysis to determine CD8 level (scale bar: 100 μm; **P < 0.01 vs. Normal group). **F** Expression correlation between METTL3 and CD8 in endometrial tissues microarray cohort by Pearson correlation analysis (*R* = 0.78, *P* < 2.2e-16). **G** The protein and mRNA expressions of METTL3 in HEC-1 A, HEC-1B, and Ishikawa cell. **H** The protein and mRNA expressions of METTL3 in Ishikawa cell with or without METTL3 overexpression (**P < 0.01 vs. Vector group). **I** The protein and mRNA expressions of METTL3 in HEC-1 A and HEC-1B cell with or without shMETTL3 (**P < 0.01 vs. shNC group). **J** The proliferation of CD8^+^ T cell in co-cultured system (**P < 0.01 vs. shNC group, ^##^*P* < 0.01 vs. Vector group). **K** The proliferation of HEC-1 A, HEC-1B, and Ishikawa cell in co-cultured system (*P < 0.05 vs. shNC group, **P < 0.01 vs. shNC group, ^##^*P* < 0.01 vs. Vector group). **L** The apoptosis of HEC-1 A, HEC-1B, and Ishikawa cell in co-cultured system (**P < 0.01 vs. shNC group, ^##^*P* < 0.01 vs. Vector group). **M** The migration of HEC-1 A, HEC-1B, and Ishikawa cell in co-cultured system (scale bar: 100 μm; **P < 0.01 vs. shNC group, ^##^*P* < 0.01 vs. Vector group). **N** Representative images of xenograft tumors in BALB/C female mice by Ishikawa cell transfected with or without METTL3 overexpression. **O** Average tumor volume of BALB/C female mice (**P < 0.01 vs. Vector group). **P** Average tumor weight of BALB/C female mice (**P < 0.01 vs.vector group). **Q** Frequency of CD8^+^ T cell in the peripheral blood of BALB/C female mice (**P < 0.01 vs. Vector group). **R** IHC staining of METTL3 and CD8 in solid tumor of BALB/C female mice (scale bar: 100 μm; **P < 0.01 vs. Vector group)

The role of METTL3 in EC was first investigated in a previous study by Liu and coworkers, who showed that reduced METTL3 expression partially contributed to the reduction in m6A methylation in EC patients. Furthermore, downregulation of METTL3 in EC led to increased proliferation and tumorigenicity of EC cells by activating the AKT pathway, suggesting an oncogenic mechanism of reduced METTL3 expression in EC, and overexpression of METTL3 could be a potential approach to treat EC [5]. However, it is unclear whether reduced METTL3 expression contributes to the progression of EC by regulating immune evasion. Our present study found that METTL3 was downregulated in EC patients (Fig. 1A-C). Furthermore, a low level of METTL3 expression was a negative prognostic indicator fthe biological characteristics of EC cells, such as cell proliferation, migration, and invasion, but limited studies have focused on the role of m6A modifications in EC immune evasor EC patients, indicating that METTL3 has the potential to be a diagnostic and prognostic biomarker for EC (Fig. 1D). In addition, there is a positive correlation between METTL3 and CD8 in endometrial tissues microarray cohort (Fig. 1E, F), METTL3 could inhibit EC progression partly by activating CD8^+^ T cells in vitro (Fig. 1G-M) and in vivo (Fig. 1O-R). This evidence indicated that enhancing METTL3-mediated immunosurveillance is an important approach for inhibiting EC. However, the exact molecular mechanism underlying METTL3-mediated immunosurveillance in EC is largely unknown.

**Fig. 2:**
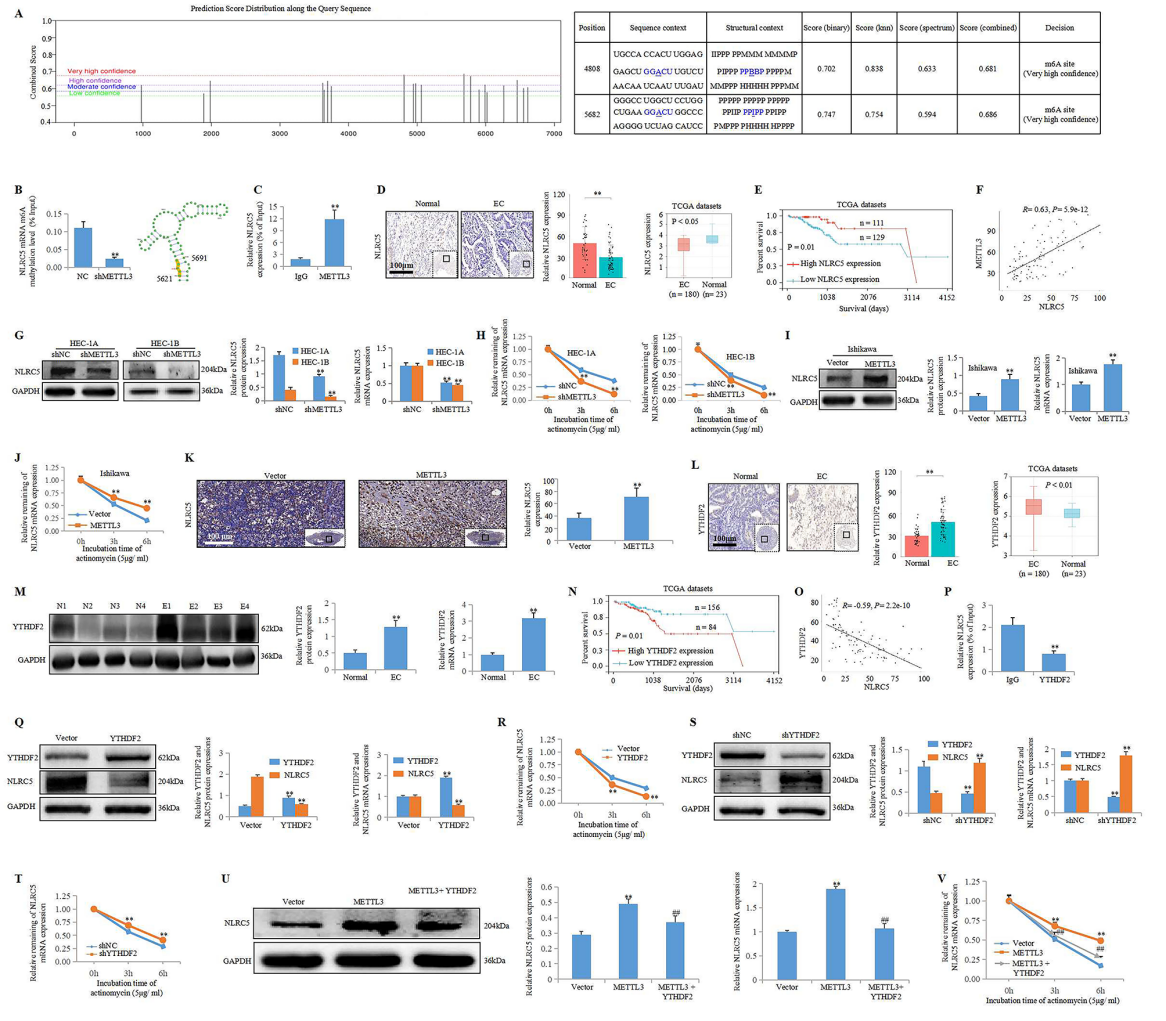
METTL3 inhibits YTHDF2-mediated NLRC5 mRNA degradation. **A** m6Avar database of m6A modification on NLRC5 mRNA sequence and the very high confidence m6A site location (4808 and 5682) on NLRC5 mRNA sequence. **B** MeRIP-PCR data in HEC-1B cell shows the relative quantity of NLRC5 mRNA immunoprecipitated by the m6A antibody (m6A-IP) and IgG in cells with or without shMETTL3 (**P < 0.01 vs. shNC group), and the NLRC5 mRNA m6A modification site. **C** RIP-PCR data in Ishikawa cell with or without METTL3 overexpression shows the content of NLRC5 mRNA immunoprecipitated by METTL3 antibody (**P < 0.01 vs. IgG group). **D** NLRC5 expression in EC form endometrial tissues microarray cohort (scale bar: 100 μm; **P < 0.01 vs. Normal group) and TCGA datasets (*P < 0.05 vs. Normal group). **E** Kaplan-Meier curves of correlation of NLRC5 with the EC cumulative survival from TCGA datasets (*P* = 0.01). **F** Expression correlation between METTL3 and NLRC5 in endometrial tissues microarray cohort by Pearson correlation analysis (*R* = 0.63, *P* = 5.9e-12). **G** The protein and mRNA expressions of NLRC5 in HEC-1 A and HEC-1B cell with or without shMETTL3 (**P < 0.01 vs. shNC group). **H** The stability of NLRC5 mRNA in HEC-1 A and HEC-1B cell with or without shMETTL3 (**P < 0.01 vs. shNC group). **I** The protein and mRNA expressions of NLRC5 in Ishikawa cell with or without METTL3 overexpression (**P < 0.01 vs. Vector group). **J** The stability of NLRC5 mRNA in Ishikawa cell with or without METTL3 overexpression (**P < 0.01 vs. Vector group). **K** IHC staining of NLRC5 in solid tumor of BALB/C female mice (scale bar: 100 μm; **P < 0.01 vs. Vector group). **L** YTHDF2 expression in EC form endometrial tissues microarray cohort (scale bar: 100 μm; **P < 0.01 vs. Normal group) and TCGA datasets (*P < 0.05 vs. Normal group). **M** The protein and mRNA expressions of YTHDF2 in endometria from normal subjects and EC patients (n = 4, **P < 0.01 vs. Normal group). **N** Kaplan-Meier curves of correlation of YTHDF2 with the EC cumulative survival from TCGA datasets (*P* = 0.01). **O** Expression correlation between YTHDF2 and NLRC5 in endometrial tissues microarray cohort by Pearson correlation analysis (*R =* -0.59, *P* = 2.2e-10). **P** RIP-PCR data shows the content of NLRC5 mRNA immunoprecipitated by YTHDF2 antibody (**P < 0.01 vs. IgG group). **Q** The protein and mRNA expressions of YTHDF2 and NLRC5 in HEC-1B cell with or without YTHDF2 overexpression (**P < 0.01 vs. Vector group). **R** The stability of NLRC5 mRNA in HEC-1B cell with or without YTHDF2 overexpression (**P < 0.01 vs. Vector group). **S** The protein and mRNA expressions of YTHDF2 and NLRC5 in HEC-1B cell with or without shYTHDF2 (**P < 0.01 vs. shNC group). **T** The stability of NLRC5 mRNA in HEC-1B cell with or without shYTHDF2 (**P < 0.01 vs. shNC group). **U** The protein and mRNA expressions of NLRC5 in HEC-1B cell with or without METTL3 overexpression or METTL3 + YTHDF2 overexpression (**P < 0.01 vs. Vector group, ^##^*P* < 0.01 vs. METTL3 overexpression group). **V** The stability of NLRC5 mRNA in HEC-1B cell with or without METTL3 overexpression or METTL3 + YTHDF2 overexpression

Next, the detailed molecular mechanisms by which METTL3 promotes immunosurveillance in EC was investigated. The innate immune molecule NLRC5 is a recently identified pattern recognition receptor (PRR) plays a leading role in the modulation of MHC class Idependent immune responses [6]. Increasing amounts of evidence indicate that NLRC5 is a target for immune evasion by numerous cancers [7]. Our previous study indicated that NLRC5 could be a potential approach to enhance antitumor immune responses in EC [8]. We wondered whether METTL3 promotes immunosurveillance in EC by regulating NLRC5. Firstly, we indicated that METTL3 could recognize m6A modification on NLRC5 mRNA (Fig. 2A-C).Then we found a low level of NLRC5 expression was a negative prognostic indicator for EC patients (Fig. 2D, E), and the expression of METTL3 was positive correlated with NLRC5 expression in endometrial tissues microarray cohort (Fig. 2F). Lastly, we suggested that METTL3could positively regulate the expression of NLRC5 and inhibit its degradation in EC (Fig. 2G-K). The fate of a target mRNA generally relies on its specific recognition by m6A readers, such as YTHDF family members; the members of the YTHDF family are some of the most common m6A readers, and they recognize m6A modifications in thousands of mRNA transcripts and regulate the stability, translation and degradation of these mRNAs, thus affecting gene expression under normal and stress conditions [9, 10]. YTHDF2 was the first identified m6A reader [11]. In our study, we found that the expression of YTHDF2 was significantly increased in EC patients (Fig. 2L, M), and a high level of YTHDF2 expression was correlated with poor prognosis in EC patients (Fig. 2N). Furthermore, YTHDF2 could recognize m6A modification on NLRC5 mRNA and promote NLRC5 mRNA degradation in EC (Fig. 2O-T). Mechanistically, METTL3 promoted the stability of NLRC5 mRNA by inhibiting YTHDF2-mediated NLRC5 mRNA degradation (Fig. 2U, V).

In summary, our study indicated that METTL3, YTHDF2, and NLRC5 have potential to be the diagnostic and prognostic biomarkers for EC. Furthermore, we revealed a novel antitumor immunosurveillance mechanism in EC. METTL3-mediated m6A modification promotes endometrial cancer immunosurveillance by activating the NLRC5 *via* a YTHDF2-dependent mechanism, suggesting that regulation of NLRC5 activation *via* METTL3-mediated m6A methylation could be clinically important in EC. Nevertheless, the sample size of EC tissues in our endometrial tissue microarray cohort analysis was relatively insufficient. Moreover, whether the METTL3/YTHDF2/NLRC5 axis could serve as an effective immunotherapeutic target in EC should also be confirmed in the future.

## Electronic supplementary material

Below is the link to the electronic supplementary material.

Supplementary Material 1

Supplementary Material 2

## Data Availability

Please contact the corresponding author for data requests.
