# Supplementary material for: METTL3 facilitates immunosurveillance by inhibiting YTHDF2-mediated NLRC5 mRNA degradation in endometrial cancer
Source: Biomark Res. 2023 Apr 21;11:43. doi: 10.1186/s40364-023-00479-4 (PMC10122371; doi:10.1186/s40364-023-00479-4)
Supplement: Supplementary file 1 — Supplementary Material 1 [file 40364_2023_479_MOESM1_ESM.docx]

**Materials and methods**

**Patients and endometrial tissue microarray**

This study was approved by the Institutional Review Board of Anhui Medical University (No: 20180023). A written informed consent was obtained from all patients prior to sample collection and storage. The tissue microarray included 60 EC endometrial tissues and 36 normal endometrial tissues; these tissues were described in our previous study [8]. Among the EC samples, 81.7% (n = 49) were collected from postmenopausal patients, and 18.6% (n = 11) were collected from premenopausal patients. A total of 85.0% (n = 51) of these samples exhibited endometrioid histology, and 15.0% (n = 9) exhibited serous histology. A total of 33.3% (n = 20) of these samples were FIGO stage I, 46.7% (n = 28) were FIGO stage II, 20.0% (n = 12) were FIGO stage III and IV, 90.0% (n = 54) were G1-G2 stage, and 10.0% (n = 10) were G3 stage. A total of 83.3% (n = 50) of these samples had ≤ 50% myometrial invasion, and 16.7% (n = 10) had > 50% myometrial invasion. A total of 10.0% (n = 6) of these patients were positive for lymphatic node metastasis, and 90.0% (n = 54) were negative for lymphatic node metastasis.

**Immunohistochemistry (IHC)**

IHC assays were performed as previously described [8]. Primary antibodies against METTL3 (15073-1-AP, Proteintech, diluted 1:100), YTHDF2 (24744-1-AP, Proteintech, diluted 1:100), NLRC5 (DF13672, Affinity, diluted 1:100), and CD8 (GB12068, Servicebio, diluted 1:3000) were used. The secondary antibody was purchased from Servicebio (G1210-2-A). Quantitative analyses were conducted for five random fields at × 200 magnification for each endometrial tissue slice. All sections were observed under an Axioscope A1 microscope (Carl Zeiss, Germany) and photographed. The background light of each photo was consistent. Dark brown staining indicated a positive reaction. The intensity of dark brown staining was analyzed using Image-Pro plus 6.0 (Media Cybernetics, Inc.).

**Cell lines and cell culture**

The human HEC-1A and HEC-1B EC cell lines were purchased from American Type Culture Collection (ATCC), and the Ishikawa EC cell line was purchased from European Collection of Authenticated Cell Cultures (ECACC). The human endometrial cancer cell lines were grown in RPMI-1640 (Invitrogen, USA) supplemented with 10% fetal bovine serum (FBS; HyClone, UT, USA) in a 5% CO_2_ incubator at 37 °C.

**Lentivirus construction and infection**

The METTL3, YTHDF2, and NLRC5 overexpression or knockdown lentivirus systems were purchased from Sangon Biotech (Shanghai) Co.,Ltd., and the lentiviral vectors carried FLAG tags and puromycin resistance genes. Empty lentivirus served as the negative control. Cells were cultured in 2 μg/mL puromycin to screen for cells with stable METTL3, YTHDF2, and NLRC5 overexpression or downregulation. Gene expression was verified by quantitative real-time PCR analysis (qRT‒PCR) and western blotting.

**Western blotting**

Western blotting assays were performed as described previously [8]. The primary antibodies used in this study included antibodies against METTL3 (15073-1-AP, Proteintech, diluted 1:1000), YTHDF2 (24744-1-AP, Proteintech, diluted 1:5000), NLRC5 (DF13672, Affinity, diluted 1:1000), and GAPDH (E-AB-20032, Elabscience, diluted 1:10000). The horseradish peroxidase-conjugated secondary antibody was from Thermo Fisher Scientific. Signals were detected using a LI-COR/Odyssey infrared imaging system (LI-COR Biosciences). The results were quantitatively analyzed using ImageJ software (National Institutes of Health).

**QRT‒PCR**

qRT‒PCR analysis was performed as described previously [8]. Transcript levels were analyzed according to the 2^-ΔΔCT^ method, and glyceraldehyde-3-phosphate dehydrogenase (GAPDH) expression was used as the control. All the primer sequences are provided as follows:

METTL3

Forward: 5’-AGCCCCACTTCCTACCAGATG-3’

Reverse: 5’-TGAGAACTGTTATTTCCCCATGC-3’

YTHDF2

Forward: 5’-AGCCCCACTTCCTACCAGATG-3’

Reverse: 5’-TGAGAACTGTTATTTCCCCATGC-3’

NLRC5

Forward: 5’-GTTCTTAGGGTTCCGTCAGCG-3’

Reverse: 5’-CAGTCCTTCAGAGTGGCACAGAG-3’

GAPDH

Forward: 5’-ACCCGCCCTATCTCAACTACC-3’

Reverse: 5’-AGGACACCATAATGACAGCC-3’

**CD8^+^ T-cell proliferation assay**

The purification of CD8^+^ T cells was performed as described previously [8]. CD8^+^ T cells were stimulated with antibodies against CD3 (300438, BioLegend) and CD28 (302934, BioLegend) and cocultured with lentivirus-infected cells in 96-well plates at a ratio of 5:1 for 3 days. The proliferation of the CD8^+^ T cells was measured by carboxyfluorescein diacetate succinimidyl ester (CFSE, S8269, Selleck, USA) using flow cytometry. The data were analyzed using FlowJo software (FlowJo™ v10.8).

**Cell counting kit-8 (CCK-8) assay**

The proliferation of lentivirus-infected EC cells in the coculture system was assessed using the CCK-8 assay (Dojindo Laboratories, Japan) as described previously [8].

**Annexin V APC/7-AAD double staining assay**

The apoptosis of lentivirus-infected EC cells in the coculture system was assessed using APC and 7-AAD-conjugated Annexin V staining (BD Pharmingen, La Jolla, CA) as described previously [8].

**Migration assay**

The migration assay was performed using Transwell chamber (Millipore, Billerica, USA) to assess the lentivirus infected EC cell in the co-cultured system. EC cells (2 × 10^4^) were plated in the upper chamber with Matrigel-coated membranes and incubated overnight. A total of 1 × 10^5^ CD8^+^ T cells were seeded in the lower chamber in medium supplemented with 10% fetal bovine serum. After 48 h, the cells were fixed in 10% neutral buffered formalin solution for 30 min and stained with 0.05% crystal violet solution for 30 min, and the number of cells that migrated through the pores to the lower surface of the membrane was counted under an inverted microscope.

**Methylated RNA immunoprecipitation quantitative PCR (MeRIP-PCR) assay**

shMETTL3 HEC-1B cells and shNC HEC-1B cells were irradiated with ultraviolet light at 254 nm and 400 mJ/cm^2^ (Stratagene Stratalinker), and the cells were lysed by disruptive sonication in RIP lysis buffer (MagnaRIP Kit; EMD Millipore) at 4 °C. Immunoprecipitation was performed overnight using an anti-m6A antibody (ab208577, Abcam) at 4 °C. After washing with PBS, RNA was extracted with phenol:chloroform isoamyl alcohol and subjected to qRT‒PCR. The amount of m6A-modified NLRC5 mRNA was determined. The relative enrichment was calculated by calculating the 2^−ΔΔCt^ value compared to the input sample. %Input = 2^−^[^Ct^_IP_ ^–^(^Ct^_Input_^−Log^_2_^10^)].

**RNA immunoprecipitation (RIP) assay**

RIP assays were performed using an EZ-Magna RIP™ RNAbinding Protein Immunoprecipitation kit (Millipore, 17-701). Cells at approximately 90% confluence were lysed with complete RIP lysis buffer supplemented with RNase inhibitor and protease inhibitor, and then, 100 μL of whole cell extract was incubated with RIP buffer containing magnetic beads conjugated to anti-METTL3 (15073-1-AP, Proteintech) or anti-YTHDF2 (24744-1-AP, Proteintech). An anti-IgG antibody (Cell Signaling Technology, 2729) was used as the nonspecific control. qRT‒PCR analysis was performed to measure the NLRC5 RNA levels. Relative fold enrichment was calculated with the 2^−ΔΔCt^ method.

**RNA stability**

To assess NLRC5 mRNA stability, cells were incubated with 5 μg/ml actinomycin D (SBR00013, Sigma) to terminate transcription. Samples were collected at 0, 3, and 6 hours after termination. NLRC5 mRNA expression was determined by qRT‒PCR.

***In* *vivo* xenograft experiments**

The experimental animal procedures were approved by the Ethics Review Committee of the Department of Laboratory Animal Science of Anhui Medical University (No: LLSC201800855). Six-week-old female BALB/C mice were purchased from Hangzhou Ziyuan Laboratory Animal Science and Technology Co. Ltd. and were housed in an SPF environment. EC cells transfected with the METTL3 overexpression or the vector (5 × 10^6^) and suspended in RPMI-1640 medium (Invitrogen, USA) were subcutaneously injected into the left armpit. After four weeks, the mice were killed, and the tumors were harvested, weighed, and imaged. The tumor volumes were calculated using the following formula: tumor volume (mm^3^) = (ab^2^)/2 [a: the longest axis (mm), b: the shortest axis (mm)]. The tumors were fixed in 4% formaldehyde, paraffin-embedded and analyzed by IHC. Peripheral blood was analyzed by flow cytometry.

**Flow cytometry for CD8^+^ T cells frequency in peripheral blood**

The CD8^+^ T cells frequency among CD45^+^ cells from the peripheral blood of mice as described previously [8]. Briefly, CD8^+^ T cells were collected and concentrated, and incubated with 5 μL of specific fluorescent CD8 antibody (100,705, Biolegend, USA) and specific fluorescent CD45 antibody (368,507, Biolegend) for 20 min. 500 μL saline was added separately into the cell solution, then centrifugation at 5000 rpm for 5 min. The supernatant was removed, and the collected cells at the bottom were dispersed into 300 μL of saline. Finally, the effective separation rate was analyzed by counting 1.2 × 10^4^ cells per sample using an LSR II flow cytometer (BD Biosciences, CA, USA) and Cell Quest software (BD Biosciences, CA, USA).

.

**Statistical analysis**

All the data were analyzed using SPSS 23.0 software (SPSS Inc., Chicago, USA). The data are expressed as the mean ± SEM. Statistical analyses were performed using analysis of variance. Significant differences and variance between groups were identified using Student’s *t* test and the F test, respectively. Correlation analysis was performed using Pearson correlation. The median METTL3 and YTHDF2 expression levels were used as cutoff values for grouping. The survival curves were measured by the Kaplan‒Meier method. *P <* 0.05 was considered statistically significant.
