# Supplementary material for: METTL3 facilitates immunosurveillance by inhibiting YTHDF2-mediated NLRC5 mRNA degradation in endometrial cancer
Source: Biomark Res. 2023 Apr 21;11:43. doi: 10.1186/s40364-023-00479-4 (PMC10122371; doi:10.1186/s40364-023-00479-4)
Supplement: Supplementary file 2 — Supplementary Material 2 [file 40364_2023_479_MOESM2_ESM.pdf]

SPRINGER NATURE

Author Services

Editing Certificate

This document certifies that the manuscript

METTL3 facilitates immunosurveillance by inhibiting YTHDF2-mediated NLRC5 mRNA degradation to activate interferon-β in endometrial cancer

prepared by the authors

Lei Zhan

was edited for proper English language, grammar, punctuation, spelling, and overall style by one or more of the highly qualified native English speaking editors at SNAS.

This certificate was issued on **November 29, 2022** and may be verified on the SNAS website using the verification code **0AFB-CD02-E58F-778A-D203** .

Neither the research content nor the authors' intentions were altered in any way during the editing process. Documents receiving this certification should be English-ready for publication; however, the author has the ability to accept or reject our suggestions and changes. To verify the final SNAS edited version, please visit our verification page at secure.authorservices.springernature.com/certificate/verify.
If you have any questions or concerns about this edited document, please contact SNAS at support@as.springernature.com.

SNAS provides a range of editing, translation, and manuscript services for researchers and publishers around the world.
For more information about our company, services, and partner discounts, please visit authorservices.springernature.com.
